# An uncommon response to metronomic therapy in a heavily pretreated patient with metastatic carcinosarcoma: a case report

**DOI:** 10.1186/s13256-016-0837-3

**Published:** 2016-03-14

**Authors:** Debora de Melo Gagliato, Rudinei Diogo Marques Linck, Regis Otaviano Franca Bezerra, Mirela Souto, Gabriel Lima Lopes, Glauco Baiocchi, Max Senna Mano

**Affiliations:** 1 Hospital Sírio Libanês, São Paulo, SP Brazil; 2 A.C. Camargo Cancer Center, São Paulo, SP Brazil

**Keywords:** Uterine carcinosarcoma, Metronomic chemotherapy, Antiangiogenic therapy

## Abstract

**Background:**

Uterine carcinosarcoma is well known for its aggressive behavior. There is little evidence regarding the gold standard combination chemotherapy in metastatic or locally advanced carcinosarcoma, due to poor survival outcomes obtained with conventional scheduled chemotherapy. This case report represents the first-ever reported objective response to a metronomic chemotherapy regimen and adds to the current literature.

**Case presentation:**

We describe a case of a Caucasian woman diagnosed with metastatic carcinosarcoma that had already been treated with multiple lines of conventional chemotherapy, with progressive disease. This patient had a surprising clinical and imaging response when treated with oral metronomic cyclophosphamide.

**Conclusions:**

We reviewed the mechanism of action implicated in metronomic chemotherapy, and correlated it with the biology of disease in carcinosarcoma. This information may add to the current literature, providing important insights to future clinical trials in this patient population.

## Background

Uterine carcinosarcoma accounts for less than 5 % of all uterine malignancies [1]. This tumor type is defined as a metaplastic malignancy similar to the biphasic components typically presented in a metaplastic breast cancer [2]. This tumor is well known for its aggressive behavior. More than half of patients present with locally advanced tumors at initial diagnosis [3, 4]. Compared with patients with uterine carcinomas, patients diagnosed with carcinosarcomas tend to have worse survival outcomes when stratified by the same matching pathologic staging [5, 6].

There is little evidence regarding the gold standard combination chemotherapy in metastatic or locally advanced carcinosarcoma, and no definitive trials have consistently proved superior overall survival advantages of one combination over another. Having mentioned that, the Gynecologic Oncology Group compared ifosfamide used either alone or combined with paclitaxel as a first-line therapy for patients with stage III or IV persistent or recurrent uterine carcinosarcoma. That group demonstrated a 31 % decrease in the risk of death (hazard ratio [HR] 0.69; 95 % confidence interval [CI] 0.49–0.97; *P* = 0.03) and a 29 % decrease in the risk of progression (HR 0.71; 95 % CI 0.51–0.97; *P* = 0.03), favoring the combination arm. Nevertheless, toxicity was also increased with combination therapy [7]. In light of this poor prognosis, aggressive behavior, and no standard chemotherapy regimen, new strategies based on biological alterations might potentially be associated with better outcomes.

In this report, we describe a case of a Caucasian woman diagnosed with metastatic carcinosarcoma that had already been treated with multiple lines of conventional chemotherapy, with little response. This patient had a surprising clinical and imaging response when treated with oral metronomic cyclophosphamide. The rationale between responses to metronomic chemotherapy in this particular rare malignancy is further discussed.

## Case presentation

Four years ago, a healthy 64-year-old Caucasian woman began to experience pain in the lower abdomen, only partially relieved by analgesics. Her past medical history was unremarkable. Magnetic resonance imaging (MRI) of the pelvis demonstrated a pelvic solid mass.

A total abdominal hysterectomy with bilateral salpingo-oophorectomy was performed. The surgical procedure also included pelvic lymphadenectomy, partial colectomy, and cystectomy. Histopathologic staging revealed a high-grade endometrial carcinosarcoma measuring 14 cm in its greatest dimension. Tumor infiltration included the uterine serosa, rectosigmoid serosa, surrounding adipose tissue, cecal appendix, right fallopian tube, and ovaries. No lymphatic or vascular invasion and no involved lymph nodes were present. Peritoneal fluid was collected and was found to be positive for malignant tumor cells. Her immunohistochemistry result was positive for CD10, vimentin, AE1/3, endomysial, and S100 antibodies and negative for actin, desmin, estrogen and progesterone receptor. The patient was given adjuvant systemic chemotherapy with six cycles of carboplatin and paclitaxel.

The patient developed local recurrence 8 months after the end of her chemotherapy and was referred for surgery. An exploratory laparotomy revealed a high-grade endometrial sarcoma involving the terminal ileum and visceral peritoneum. The patient underwent a secondary cytoreductive surgery with no residual disease. After surgery, she received two cycles of ifosfamide at 2 g/m^2^ D1-3 combined with epirubicin at 50 mg/m^2^ D1-2 every 21 days. Treatment was interrupted due to encephalopathy and hematologic toxicity. Hematologic toxicity included grade 4 neutropenia according to the National Cancer Institute (NCI) Common Terminology Criteria for Adverse Events (CTCAE) version 4.0. The patient developed symptoms of agitation, confusion, and seizure due to ifosfamide-related encephalopathy. Her neurologic symptoms were totally reversed after chemotherapy interruption.

The patient was followed for 8 months, when fluorodeoxyglucose positron emission tomography–computed tomography (FDG-PET/CT) revealed two major perihepatic peritoneal nodules near segment VI, along with several other, smaller peritoneal implants. She had a complete tertiary cytoreduction that included resection of hepatic implants near segment VI, mesentery, and peritoneal bladder. Her pathology results confirmed carcinosarcoma, with a predominant high-grade, undifferentiated stromal component. Following surgery, she received carboplatin at a target carboplatin area under the time–concentration curve of 5, combined with docetaxel at 60 mg/m^2^; however, after the third cycle, she developed grade 3 NCI CTCAE version 4.0 infusion-related allergy to carboplatin, and the fourth cycle included docetaxel monotherapy at 60 mg/m^2^.

Five months later, MRI and FDG-PET/CT demonstrated a peritoneal recurrence near hepatic segments VIII and VI, as well as the precaval lymph node and hepatic hilum with extension to the retropancreatic space. Nevertheless, a new attempt at cytoreductive surgery was ruled out. She was started on topotecan at 1.25 mg/m^2^ D1-5 every 21 days, but she developed progressive disease shortly after the fourth cycle. Her clinical condition included jaundice, malaise, and a great deterioration of PS.

**Fig. 1:**
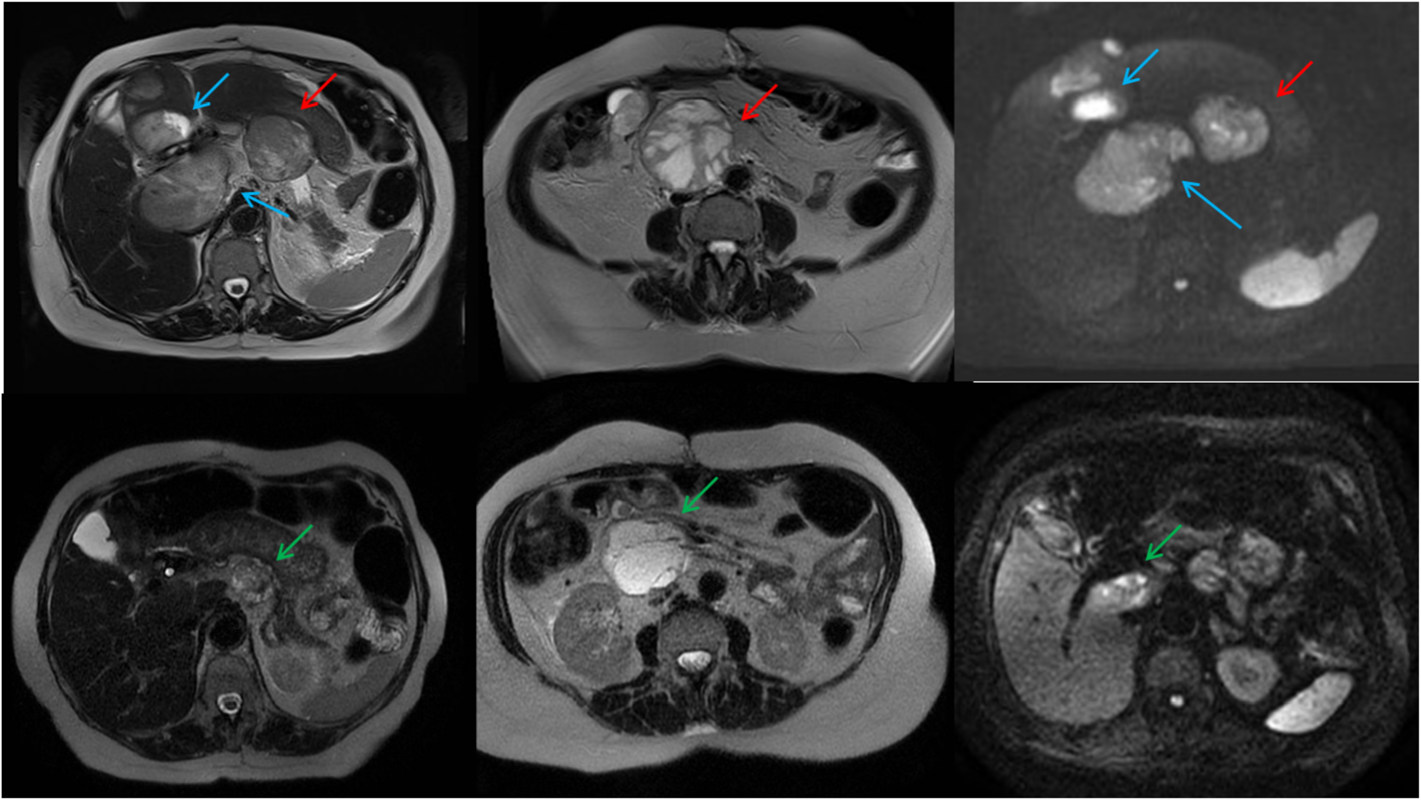
T2- and diffusion-weighted magnetic resonance imaging scans (b = 600). In the *top row*, *blue arrows* indicate hepatic lesions and *red arrows* indicate abdominal lymphadenopathy at baseline. In the *bottom row*, scans taken after metronomic chemotherapy treatment show a major reduction at the hepatic lesions (*green arrows*) as well as at the abdominal lymphadenopathy

Biliary stents were placed. The procedure was successful, and the patient experienced considerable clinical improvement. The physician team decided to initiate metronomic chemotherapy. Oral cyclophosphamide at an empiric dose of 50 mg/day, given continuously, was started. The patient tolerated this regimen quite well, started to feel better, and recovered her performance status. Abdominal MRI was performed to evaluate treatment efficacy, and the patient was found to have a partial response according to Response Evaluation Criteria in Solid Tumors (RECIST) 1.1 criteria. Figure 1 displays MRI scans obtained before and after metronomic therapy.

Three months later, the patient complained of a new abdominal pain, and MRI demonstrated a new liver mass that was interpreted as progressive disease. Metronomic therapy was switched to a standard vinorelbine dosing schedule, namely 25 mg/m^2^ D1 and D8 every 21 days. After three cycles, the patient was experiencing worsening of her abdominal pain as well as daily fever. Chemotherapy was discontinued. A new abdominal MRI scan demonstrated that the liver lesion was bigger, with a central necrotic aspect. Percutaneous drainage of the lesion confirmed that there was a hepatic abscess. After the procedure and antibiotic treatment, the patient’s condition improved. This suggested that the medical team had misinterpreted computed tomographic (CT) scans and that the new liver lesion which had developed while the patient was on metronomic chemotherapy was a liver abscess. Earlier this year, cyclophosphamide was reinitiated, and a new MRI performed 3 months after treatment initiation revealed a partial response based on RECIST 1.1. The patient has continued with oral cyclophosphamide therapy through the time of this report. Tomographic evaluation was performed in December 2015, and the images demonstrated a partial response in comparison to the initial CT scans performed before cyclophosphamide treatment. The patient’s progression-free survival is 22 months at the time of this report.

## Discussion

In this case report, we described a heavily pretreated patient diagnosed with carcinosarcoma who experienced an objective response to metronomic therapy. The biologic rationale involved in this response might be related to a high dependence on vascular growth factors for disease progression. It is import to emphasize that optimal dosing and scheduling of metronomic chemotherapy remains a challenging subject, with no data derived from randomized controlled trials to guide precise, optimal scheduling. We chose to provide our patient the empiric dosing of 50 mg/day of cyclophosphamide continuously.

The more aggressive clinicopathologic characteristics observed in uterine carcinosarcomas might be supported by the high degree of angiogenic activity in these tumors. Vascular endothelial growth factor (VEGF), produced by cancer cells, is considered to be a critical activator of vascular endothelial cells, and its expression has been associated with increased angiogenesis in many human tumors, including gynecologic malignancies [2, 8, 9]. Of note, the largest analysis of VEGF protein expression in uterine carcinosarcoma was performed by Emoto *et al*., who demonstrated by *in situ* hybridization high levels of VEGF expression in 35 primary uterine carcinosarcomas [8]. Other researchers also have demonstrated high levels of immunohistochemical staining of VEGF in these tumors, especially at the mesenchymal component. Additionally, VEGF staining has been associated with poor survival outcomes [2, 9, 10].

Uterine carcinosarcoma also was found to display a specific microRNA (miRNA or miR) signature in at least two different studies. Compared with benign endometrium or with epithelial uterine tumors (endometrioid or papillary serous), uterine carcinosarcomas are a biologically distinct disease, further supporting the hypothesis that these tumors require a therapeutic strategy distinct from those used for other epithelial tumors [11, 12]. Certain miRNAs appear to be consistently altered in the carcinosarcomas. In fact, miR-20b, a known regulator of VEGF, is upregulated in carcinosarcoma cells. miR-20b has been described as playing an important oncogenic role in solid malignancies, and its high expression is associated with tumor cell growth. Comparatively, low expression of miR-20b inhibits tumor cell growth and is associated with resistance to apoptosis in hypoxic conditions by upregulation of tumor cell VEGF [13, 14]. Thus, a possible reason to maintain tumor adaptation to hypoxia is through low expression of VEGF, as tumor stromal cells may act as a source of VEGF [14].

The concept of metronomic chemotherapy is based on a treatment that is delivered more frequently and with low daily doses. Cyclophosphamide is the most widely explored agent in such an approach. Its mechanism of action is regarded as primarily antiangiogenic in nature, affecting both the endothelial cells of tumor-supplying blood vessels and the circulating endothelial progenitor cells. In addition, recent evidence points to the presence of immunomodulatory antitumor activities in metronomic therapy. However, the importance of such immunologic and other direct anticancer cells effects, such as the interference with the hypoxia-induced factor 1α pathway, along with targeting cancer stem cells, remains undetermined [15–18]. Retrospective studies and numerous phase II clinical trials have been published in diverse clinical scenarios, mainly in patients with highly pretreated advanced tumors. Also, metronomic chemotherapy was an attractive strategy in our patient, whereas it was associated with a very favorable toxicity profile and less financial cost [15, 18].

Antiangiogenic therapies in uterine carcinosarcoma were previously evaluated *in vitro* and *in vivo* in preclinical studies, with promising results [19, 20]. In the clinical setting, different antiangiogenic drugs were tested in metastatic disease. Aflibercept, pazopanib, and thalidomide demonstrated activity, which suggests the concept that angiogenesis is an important pathway in carcinosarcoma development [21–23]. Several other targeted strategies have now been developed [2, 24].

## Conclusions

Uterine carcinosarcoma is a rare and aggressive disease, with no standard treatment, especially in a recurrent or progressive disease setting. In this case report, we review the mechanism of action implicated in metronomic chemotherapy, exploring an antiangiogenic strategy in a patient diagnosed with advanced carcinosarcoma. More details are necessary to establish a standard treatment, but this report adds to the current literature.

## Consent

Written informed consent was obtained from the patient for publication of this case report and any accompanying images. A copy of the written consent is available for review by the Editor-in-Chief of this journal.

## Abbreviations

CI: confidence interval
CT: computed tomographic
CTCAE: Common Terminology Criteria for Adverse Events
FDG-PET/CT: fluorodeoxyglucose positron emission tomography–computed tomography
HR: hazard ratio
miRNA: microRNA or mIR
MRI: magnetic resonance imaging
NCI: National Cancer Institute
RECIST: Response Evaluation Criteria in Solid Tumors
VEGF: vascular endothelial growth factor
